# Crotamiton-loaded tea tree oil containing phospholipid-based microemulsion hydrogel for scabies treatment: *in vitro*, *in vivo* evaluation, and dermatokinetic studies

**DOI:** 10.1080/10717544.2021.1979131

**Published:** 2021-09-25

**Authors:** Lihua Chen, Majed Alrobaian, Obaid Afzal, Imran Kazmi, Sunil K. Panda, Abdulmalik Saleh Alfawaz Altamimi, Fahad A. Al-Abbasi, Waleed H. Almalki, Hanadi A. Katouah, Tanuja Singh, Kriti Soni, Abdul Hafeez, Sarwar Beg, Vikas Kumar, Mahfoozur Rahman

**Affiliations:** a Department of Dermatology, Taizhou People’s Hospital, Taizhou, China; b Department of Pharmaceutics and Industrial Pharmacy, College of Pharmacy, Taif University, Taif, Saudi Arabia; c Department of Pharmaceutical Chemistry, College of Pharmacy, Prince Sattam Bin Abdulaziz University, Al-Kharj, Saudi Arabia; d Department of Biochemistry, Faculty of Science, King Abdulaziz University, Jeddah, Saudi Arabia; e Menovo Pharmaceuticals Research Lab, Ningbo, People's Republic of China; f Department of Pharmacology and Toxicology, College of Pharmacy, Umm Al-Qura University, Makkah, Saudi Arabia; g Chemistry Department, Faculty of Applied Sciences, Umm Al-Qura University, Makkah, Saudi Arabia; h Department of Botany, Patliputra University, Patna, India; i Formulation Development, Dabur Research Foundation, Ghaziabad, India; j Glocal School of Pharmacy, Glocal University, Saharanpur, India; k Department of Pharmaceutics, School of Pharmaceutical Education and Research, Jamia Hamdard, New Delhi, India; l Department of Pharmaceutical Sciences, Shalom Institute of Health & Allied Sciences, Sam Higginbottom University of Agriculture, Technology & Sciences, Allahabad, India

**Keywords:** Crotamiton, microemulsion, pseudo-ternary phase diagram, scabies, *in vitro* drug release, topical availability, novel drug delivery

## Abstract

Crotamiton (CRT) is a commonly approved drug prescribed for the scabies treatment in many countries across the globe. However, poor aqueous solubility and low bioavailability, and side effects restrict its use. To avoid such issues, an appropriate carrier system is necessary which can address the aforementioned challenges for attaining enhanced biopharmaceutical attributes. The current study intends to provide a detailed account on the development and evaluation of CRT-loaded microemulsion (ME) hydrogel formulation containing tea tree oil (TTO) for improved drug delivery for scabies treatment in a safe and effective manner. Pseudo-ternary phase diagrams were constructed with TTO as the oily phase, and Cremophor^®^EL was used as the surfactant in a mass ratio 2:1 with co-surfactants (mixture of phospholipid 90G and Transcutol^®^P), and aqueous solution as the external phase. The optimized drug-loaded ME formulation was evaluated for skin penetration, retention, compliance, and dermatokinetics. The nonirritant behavior of the formulation was revealed by skin histopathology, which showed no changes in normal skin histology. In comparison to the conventional product, dermatokinetic experiments revealed that CRT has greater penetration and distribution in the epidermis of the mice skin. The findings imply that the proposed lipid-based ME hydrogel can aid in the resolution of CRT issues by providing a better and safer delivery option to epidermis and deeper epidermis in substantial quantities.

## 1. Introduction

Several studies have documented the use of poorly water soluble (or hydrophobic) drugs in topical liquid/semi-solid formulations (Das et al., 2012). Encapsulating hydrophobic drugs in polymeric nanoparticles (Das et al., 2012), lipid-based nanoparticles (Das et al., 2012), nanoemulsion, or liposomes before adding them to liquid/semi-solid formulations greatly helps in improving their delivery across the skin barriers. Microemulsion (ME) is an option to load poorly water-soluble drugs into liquid/semi-solid formulations (Froelich et al., 2017). The solubility of hydrophobic drugs can be improved via ME (Froelich et al., 2017). Unlike conventional emulsion, ME is a thermodynamically stable system with droplet sizes ranging from 10 to 100 nm and exhibits longer shelf-life (Froelich et al., 2017). It is also optically clear and macroscopically homogenous (Froelich et al., 2017). Furthermore, considerable energy input is not required for production, thus preparation is simple to produce and scale up, making it cost-effective (Benbow & Campbell, 2019). ME is made up of oil, aqueous phase, and the surfactant (a combination of surfactant and co-surfactant) (Benbow & Campbell, 2019). It can solubilize weakly water-soluble drugs and enhance penetration of the drugs through the skin, hence increase their bioavailability (Farghaly et al., 2018). By encapsulating therapeutic molecules in nano-droplets, ME can also improve drug stability (Farghaly et al., 2018). As ME is difficult to apply to the skin due to their great fluidity, a gelling agent is commonly used to increase their viscosity (Farghaly et al., 2018). In such cases, topical ME-based formulations with strong skin penetration capacity are likely to outperform conventional emulsion-based cream formulations (Das et al., 2020). Such formulations may also minimize the amount of CRT needed compared with commercially available CRT topical cream (Mila-Kierzenkowska et al., 2017).

Scabies is a parasite disease which affects humans and animals. This is caused by the *Sarcoptes scabiei*, a microscopic parasite that is typically not apparent (Mila-Kierzenkowska et al., 2017). Scabies is found worldwide and is a major public health issue in developing countries. It is reported to infect around 300 million people each year worldwide (Mila-Kierzenkowska et al., 2017) and affects people of all ages, genders, races, and social classes; nevertheless, poverty, malnutrition, homelessness, dementia, and poor hygiene are key risk factors (Mila-Kierzenkowska et al., 2017). Direct skin-to-skin contact with an infected person is required for transmission of the mite to another person, and it usually requires 15–20 minutes of close contact (Mila-Kierzenkowska et al., 2017). Pruritus with nocturnal aggravation and scabietic nodules are clinical features of human scabies, and visible skin burrows can represent scabies pathognomonic lesions (Alexander, 1984; Mila-Kierzenkowska et al., 2017). Scabies mites burrow into the human epidermis and are widely thought to reside in the lower stratum corneum with their gnathosoma embedded into deeper layers of the epidermis (Alexander, 1984). Scabies is widely treated with several drugs known as acaricides, but the best treatment is still up for debate, and the search for the perfect scabicide continues (Alexander, 1984). The treatment of scabies includes crotamiton (CRT) 10% cream, permethrin 5% cream, benzyl benzoate (BB) 10% and 20% lotion or emulsion, lindane 1% lotion or cream, ivermectin 0.8% cream (Mila-Kierzenkowska et al., 2017). The majority of existing scabicides are potentially dangerous, with mild to severe cutaneous and systemic adverse effects (Alexander, 1984). The safety of ivermectin (the only oral treatment for scabies) in the elderly, individuals with liver disease, children under the age of five, and pregnant women has not been sufficiently proven. Adults have utilized BB extensively, as well as infants, babies, and breast-feeding mothers in diluted form (Alexander, 1984). *In vitro* data show that BB is superior to permethrin at killing scabies mites, and that it is more effective in severe crusted scabies (Mila-Kierzenkowska et al., 2017). Initial acute burning sensation after local application of strong lotion (25% v/v) is the most prevalent adverse event connected with the use of BB (Mila-Kierzenkowska et al., 2017). Further side effects include blister formation, crusting, itching, leaking, reddening, and scaling of skin after repeated use (Mila-Kierzenkowska et al., 2017). Mostly, oral treatment therapeutics failed in treatment of scabies, due to secondary applications and adverse effects (Mila-Kierzenkowska et al., 2017). Crotamiton (crotonyl-N-ethyl-o-toluidine) as 10% lotion or cream is approved for use in adults with scabies. However, its poor solubility, poor bioavailability and side effects, restricted their use (Mila-Kierzenkowska et al., 2017). To avoid these issues, a suitable carrier system that can transport this potential therapeutic molecule and deliver to the site is the need of the hour. Tea tree oil (TTO) has most potent active ingredient terpinen-4-ol, and as oil phase, TTO has showed promise as an acaricide (Tighe et al., 2013). Their therapeutic benefits as formulation reported in wide range of dermatological disorders. It is developed as a drug in an appropriate pharmaceutical base (cream, ointment, or gel) in various concentrations (Tighe et al., 2013). However, it has drawbacks, such as increased volatility due to which 90% of TTO evaporates very quickly from the skin surface, limiting TTO dermal penetration into the deeper skin layers (Hadaś et al., 2017). *In vitro* study of TTO against human scabies mites revealed a superior outcome (60 minutes median survival time with 5% TTO) as compared to standard treatments (150 minutes with ivermectin 100 g/g; 120 minutes with permethrin 5%) (Alexander, 1984; Mila-Kierzenkowska et al., 2017). TTO has also been utilized as a regular supplementary treatment for crusted scabies (Royal Darwin Hospital, treatment protocol) in conjunction with BB and oral ivermectin (Alexander, 1984; Mila-Kierzenkowska et al., 2017). As a result, the primary goal of this work was to develop CRT-loaded ME-based hydrogel containing TTO for safe and effective drug delivery application against scabies. ME area was determined using pseudo-ternary phase diagrams created with various surfactant and co-surfactant compositions. Then, based on the ME zones in the phase diagrams, CRT-loaded TTO ME and ME gels were produced and investigated. ME's stability was also tested over a period of six weeks under various storage circumstances. Finally, the CRT-loaded TTO ME gel formulations were subjected to an *in vitro* membrane penetration (or release) test, which was compared with Crotorax 10% cream (Abbott Healthcare Pvt. Ltd., Mumbai, India).

## 2. Materials and methods

### 2.1. Materials

Polyoxyethylene castor oil (Cremophor^®^EL) was purchased from BASF SE (Ludwigshafen, Germany); highly purified diethylene glycol monoethyl ether (Transcutol^®^P) was obtained from Gattefossé (Saint-Priest Cedex, France); CRT was purchased from Sigma Aldrich (St. Louis, MO). Lipoid GmBH (Köln, Germany) provided free Phospholipon 90G samples. From S.D. Fine Chemicals Ltd. (Mumbai, India), Tween 20, Tween 80, Span 20, and Span 80 were purchased. Absolute ethanol was purchased from Bengal Chemicals Ltd. (Kolkata, India). Pluronic^®^F-127 was procured from BASF Corporation (Florham Park, NJ). In this work, all other compounds were of analytical quality, and double distilled water was used.

### 2.2. Analytical method development

CRT was separated chromatographically using the high-performance liquid chromatography (HPLC) method. Method development was carried out using an Agilent 1260 Infinity HPLC system with a Zorbax C18 column and isocratic elution of a mobile phase comprising a mixture of ethanol, water, and formic acid (30:40:30%, v/v). At a flow rate of 0.5 mL/min, the detecting wavelength was set at 275 nm. The run time was set to eight minutes, while retention time for the drug was observed as 6 min. For accuracy, precision, robustness, limit of quantification (LOQ), and limit of detection (LOD), the developed method's linearity range was examined and confirmed.

### 2.3. Pseudo-ternary phase diagrams

Pseudo-ternary phase diagrams were created using the aqueous titration method at 37 °C. Based on the solubility, Cremophor^®^EL was chosen as the surfactant in a mass ratio of 2:1 with the co-surfactant (a mixture of phospholipid and Transcutol^®^P in a mass ratio of 1:4). TTO was utilized as the oil phase, and various clear ratios of oil and *S*_mix_ (a mixture of surfactant and co-surfactant ranging from 1:9 to 9:1) were titrated against water until turbidity or haziness developed. Various aqueous phase and *S*_mix_ ratios were also developed and titrated against oil in the same way. After all, as shown in Table 1, three distinct phase diagrams were generated with varied ratios of surfactant to cosurfactant (*S*_mix_) ranging from 1:1 to 3:1. The physical condition of the ME was illustrated by one axis representing the aqueous phase, the other representing the oil, and the third indicating the fixed *S*_mix_ ratio on pseudo-ternary diagrams. The ME region was described in the diagram as the area where visual inspection provided clean and transparent formulations (Brand et al., 2002; Golwala et al., 2020).

**Table 1.** The pseudo-ternary phase diagrams on the different ratio of surfactant:cosurfactant used.

S. no.	Pseudo-ternary diagram code	*S*_mix_
1	Figure 1(A)	Cremophor^®^EL and cosurfactant (1:1)
2	Figure 1(B)	Cremophor^®^EL: cosurfactant (2:1)
3	Figure 1(C)	Cremophor^®^EL: cosurfactant (3:1)

Cosurfactant: Transcutol^®^P and phospholipid (PL90G); *S*_mix_: surfactant to cosurfactant.

### 2.4. Selection of microemulsion based on pseudo-ternary phase diagrams

The ME zone was identified using pseudo-ternary phase diagrams. Different formulations/points were chosen from each diagram based on the least amount of emulsifier utilized in the formulations.

### 2.5. Development of microemulsion

In a nutshell, the non-aqueous phase containing phospholipid, Transcutol^®^P, and Cremophor^®^EL was heated to 50–60 °C for 15 minutes with constant stirring to obtain a homogeneous surfactant and phospholipid solution (Golwala et al., 2020). The temperature of the aforesaid system was gradually lowered, and CRT (equal to 0.1% w/w) was introduced to the TTO (16% w/w) as an oily phase. A transparent yellow ME was obtained by progressively adding the needed amount of aqueous phase while stirring continuously.

### 2.6. Characterizations

Six ME formulations were produced and described for the parameters listed below, based on the ME areas found in the phase diagrams.

#### 2.6.1. Emulsion attributes

The formulations were stored at 37 °C for 42 days at random to examine the product's sustenance of its characteristics. The formulations were examined for creaming, cracking, and phase separation, among many other macroscopic characteristics.

#### 2.6.2. Drug content

The amount of drug present in the developed systems was evaluated by extracting the drug in ethanol and then evaluating it with a HPLC analysis as per the method reported in section 2.2.

#### 2.6.3. Micromeritics and zeta potential

Using the dynamic light scattering (DLS) technique, the Malvern Zetasizer Nano ZS (Malvern Instruments, Worcestershire, UK) was utilized to determine the particle size (*z*-average diameter) and polydispersity index (PDI) of the ME formulations at 25 °C (Tian et al., 2020). The MEs' size and PDI were determined without diluting the samples because water dilution would damage the ME structure. A glass cuvette was filled with ME. The cuvette was then placed in the Zetasizer Nano ZS cuvette holder and analyzed using DTS v 6.12 software (Malvern Instruments, Worcestershire, UK). Using the same equipment, the zeta potential of the formulations was measured. The mean result was calculated using the values of three successive observations.

#### 2.6.4. Shape of globules

At the Central Instrumentation Laboratory (CIL), Panjab University (Chandigarh, India), photo microscopy and transmission electron microscopy (TEM) were used to determine the morphology and structure of the globules of drug-loaded ME, and microphotographs were taken at appropriate magnifications.

#### 2.6.5. Measurement of pH

The topical nature of the formulations, pH measurements were required to guarantee that they were nonirritating. Using an L1-120, Cyber Scan 510 pH meter, the pH of the undiluted mixtures was determined (Eutech Instruments Pvt. Ltd., Singapore).

#### 2.6.6. Refractive index (RI)

An Abbe Refractometer (Shijiazhuang Optical Instrument Factory, Xiamen, China) was used to estimate the optical density of the constructed system at room temperature. RI of the formulation was calculated using water as a reference.

### 2.7. Development of microemulsion gel

To make the ME rheological suitable for topical applications, carbopol 971P was used to gel them. Carbopol 971P (1% w/v) was dissolved in aqueous phase for 24 hours at 4 °C, then the aqueous phase was mixed with the oily phase to make ME gel.

### 2.8. *In vitro* permeation studies

Mice excised abdomen skin was used in the permeation investigations (Kim et al., 2017). The animals were sacrificed through cervical dislocation. Surgical scissors were used to take the skin off the animal, and surgical blade no. 24 was used to remove the hair from the split skin. A vernier caliper was used to measure the thickness of the skin, and a dye test was conducted to determine the skin's integrity. The removed skin was then cleaned to remove any remaining fat and rinsed with PBS at pH 7.4 to finish. For later usage, the cleansed skin was wrapped in aluminum foil and stored in a deep freezer at −30 °C (Al-Mahallawi et al., 2019; Berthet et al., 2020). A methylene blue dye test was done to check the skin integrity prior to completing the *in vitro* skin permeation investigations (Berthet et al., 2020). After that, the Franz diffusion cells were put to an *in vitro* skin permeation test (Perme Gear, Inc., Hellertown, PA). Furthermore, the excised skin was attached between the donor and receptor compartments, covering a 2 cm^2^ effective surface area with a sink capacity of 20 mL (Berthet et al., 2020). The receptor compartment, on the other hand, was filled with ethanol as a permeation medium to keep the sink condition and the temperature at 37 ± 0.5 °C. Following that, the developed formulation (1 g; equivalent to 0.1 mg of drug) was used on the skin of the donor compartment. Apart from this, CRT solution (containing 1 mg) was applied onto the skin of donor compartment for the comparison purpose. Furthermore, the applied formulation was evenly dispersed with the flat end of a spatula to keep the formulation intact with the skin for a longer period of time (Berthet et al., 2020). To maintain the receptor compartment volume, an aliquot of 2 mL of sample was withdrawn through the sampling port a set time period (0–24 h) and replaced with an equivalent amount of sink medium (Berthet et al., 2020). After making adequate dilutions, the samples were analyzed by HPLC.

### 2.9. Skin drug retention studies

The skin tissue put on the diffusion cell was carefully removed after the skin permeation experiments were completed and washed three times with distilled water to remove any leftover formulation. Furthermore, the skin was dried and weighed under the fold of tissue paper. The skin was further mashed with tissue homogenizer after drying. Following that, the obtained homogenate suspension was diluted with ethanol. For CRT extraction, a 20 mL of ethanol with chloroform combination (3:1 v/v) was agitated for two hours at 37 °C. The supernatant was filtered through a 0.45 m membrane filter (Millipore, Billerica, MA) and the drug content was quantified using the HPLC technique.

### 2.10. Dermatokinetic modeling

In the dermatokinetic studies, mice excised skin was employed. Additionally, the skin tissue was processed as described in Section 2.8, and the technique was followed as described in Section 2.9. At the proper sampling periods, the entire skin was removed from the Franz cell and washed three times to remove any adhering formulation (0–6 h). To facilitate epidermal detachment from dermis, the cleansed skin was immersed in hot water (60 °C) for 30 seconds. Both pieces were chopped into small pieces and macerated for 24 hours in a 5 mL chloroform–methanol mixture (2:1 v/v) to extract all of the drugs. After filtration over a membrane (0.45 μm), the filtrate was tested using HPLC. The acquired data were fitted into a one-compartment model using the following equation:

(1) $$C_{\text{Skin}}=\frac{K_{p}.C_{\text{max}}^{\text{Skin}}}{\left(K_{p}-K_{e}\right)}(e^{-K_{\text{pt}}}-e^{-K_{\text{et}}})$$

where *C*_Skin_ is the concentration of the drug found in epidermis or dermis at time *t*, $C_{\text{max}}^{\text{Skin}}$ is the maximum concentration received in epidermis or dermis; *K*_e_ is the elimination constant in the skin, *K*_p_ is the permeation constant in dermis. With the help of win-Nonlin Ver 5.0 software, various dermatokinetic parameters such as *K*_p_, $C_{\text{max}}^{\text{Skin}},$ *K*_e_, $T_{\text{max}}^{\text{Skin}}$ (time required to achieve $C_{\text{max}}^{\text{Skin}}$) and area under the curve (AUC_0–12 h_) were calculated using the Wagner-Nelson method (Kim et al., 2017).

### 2.11. Skin compatibility studies

The study was carried out in Animal house facility of SS Hospital and Research Institute (Patna, India) in collaboration with T.P.S. College (Patna, India). The mice (35–45 g) were housed in the stainless-steel wire cages at a temperature of 25 ± 1 °C, humidity (56 ± 5%), and lighting (12 h light/dark cycle). Food and tap water were provided *ad libitum*. All animal experiments were carried out as per the CPCSEA guidelines with approval number 1840/PO/ReBi/S/15/CPSCEA. Mice were categorized into two groups, each group containing six animals. Surgical scissors were used to remove the animal's fur, and hair was taken from the secluded skin using an operating blade no. 24. Further, the skin was cleansed three times with a saline-soaked cotton swab before applying ME gel topically (1 g equivalent to 0.1 mg of CRT) to the skin in a 5 cm^2^ region once a day for 10 days. Moreover, group 1 and group 2 mice received saline and acts as the control and ME (K7) gel, respectively. Before applying the next dose, the intact formulation was gently removed from the skin and swabbed with saline-soaked cotton. The mice were sacrificed by cervical dislocation at the end of the research, and skin samples were taken and preserved in a 10% formalin solution. Further, the acquired skin samples were stained with hematoxylin and eosin, and the histopathological changes in the skin samples were seen microscopically (Shaker et al., 2020).

### 2.12. Stability studies

All the developed ME was kept for stability studies and provided at various temperatures such as 4 ± 2 °C, 30 ± 2 °C, and 40 ± 2 °C for 42 days in closed containers. The drug content was assessed at regular intervals using the method described in drug content studies.

### 2.13. Statistical data analysis

The data were statistically evaluated using a one-way ANOVA, followed by a *post hoc* analysis using Student's *t*-test. A statistically significant result was assumed to have occurred at the level of *p*<.05.

## 3. Results and discussion

### 3.1. Screening of oils, surfactants, and co-surfactants for ME

**Figure 1.**
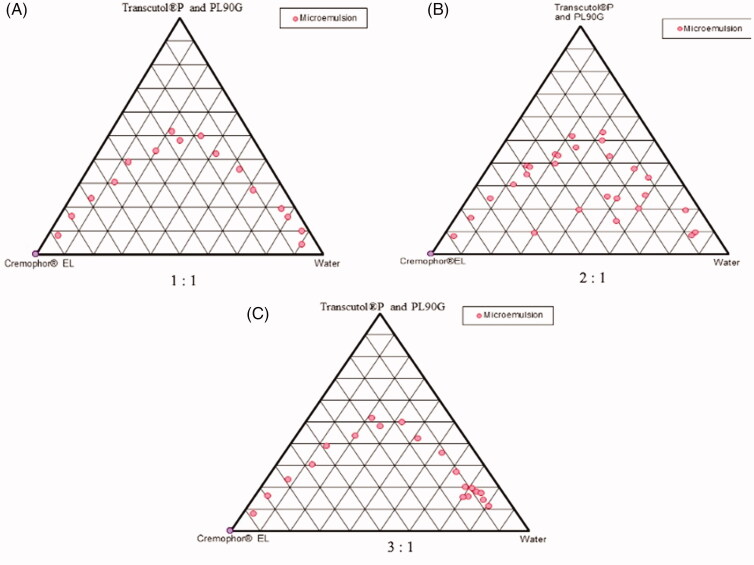
Pseudo-ternary phase diagrams shown at different *S*_mix_ at: (A) 1:1, (B) 2:1, and (C) 3:1.

The selection of the oil phase is an important consideration, in this study, TTO was used as oil phase because it possesses anti-scabies action and employed in the concentration range of 11–17%. Apart from this, Cremophor^®^EL was selected as surfactant in the mass ratio of 2:1 with the co-surfactant (mixture of Transcutol^®^P and phospholipid (PL90G)). Besides, it has also been reported to exert a cosolvent action (Radwan et al., 2017). Figure 1 shows the pseudo-ternary phase diagram which was clearly vivid Cremophor^®^EL and offered better emulsification region over other used nonionic surfactants. Furthermore, no problems of miscibility of the drug with the selected surfactant/cosurfactant observed. However, on the basis of maximum solubility and better emulsification, Cremophor^®^EL was opted as surfactant, and the required dose of CRT can be loaded into oil was 16%, due to the use of this emulsifier. Cremophor^®^EL is a well-known nonionic surfactant with improved emulsification capabilities and is biocompatible (Radwan et al., 2017). Apart from this, various literature reports have indicated the use of Cremophor^®^ EL and incorporated into the ME which enhances the skin permeation of drugs (Amiri-Rigi & Abbasi, 2019). On the other hand, the addition of a co surfactant is not always essential, as it reduces interfacial tension, which improves ME stability and biocompatibility (Amiri-Rigi & Abbasi, 2019). In this study, Transcutol^®^P and PL90G were used as co-surfactant, the presence of PL90G exploited the biocompatibility and enhances the permeation enhancement from Transcutol^®^P (Radwan et al., 2017; Amiri-Rigi & Abbasi, 2019). The presence of Transcutol^®^P reduces the stiffness of PL90G condensed films, which is essential for the production of ME globules (Radwan et al., 2017; Amiri-Rigi & Abbasi, 2019). Furthermore, the presence of a cosurfactant reduced the interface's bending stress, giving it enough flexibility to form various curvatures, which must form ME with various compositions (Radwan et al., 2017; Amiri-Rigi & Abbasi, 2019).

### 3.2. Development of ternary phase diagram and selection of formulations

A phase diagram can be used to determine the relationship between a mixture's phase behavior and its composition (Golwala et al., 2020). The phase diagrams shown in Figure 1(A–C), reflect all of the *S*_mix_ compositions. Hundreds of ME formulations may be made from a single-phase diagram, but there are no reports on the basis for choosing various formulations from a phase diagram (Golwala et al., 2020). The formulations were all chosen from pseudo-ternary phase diagrams 1B. The points were chosen based on the oil range of 11–17%. Further, the selected formulation with their codes has been shown in Table 2.

**Table 2.** The compositions of the various selected microemulsions.

Microemulsion code	% of oil	% of aqueous phase	% of * S*_mix_
Formulation from 1B pseudo-ternary phase diagrams			
K7	16	30	54
K10	13	29	58
K12	17	26	57
K15	12	30	58
K13	11	34	55
K5	14	28	58

### 3.3. Microemulsion characterization

**Figure 2.**
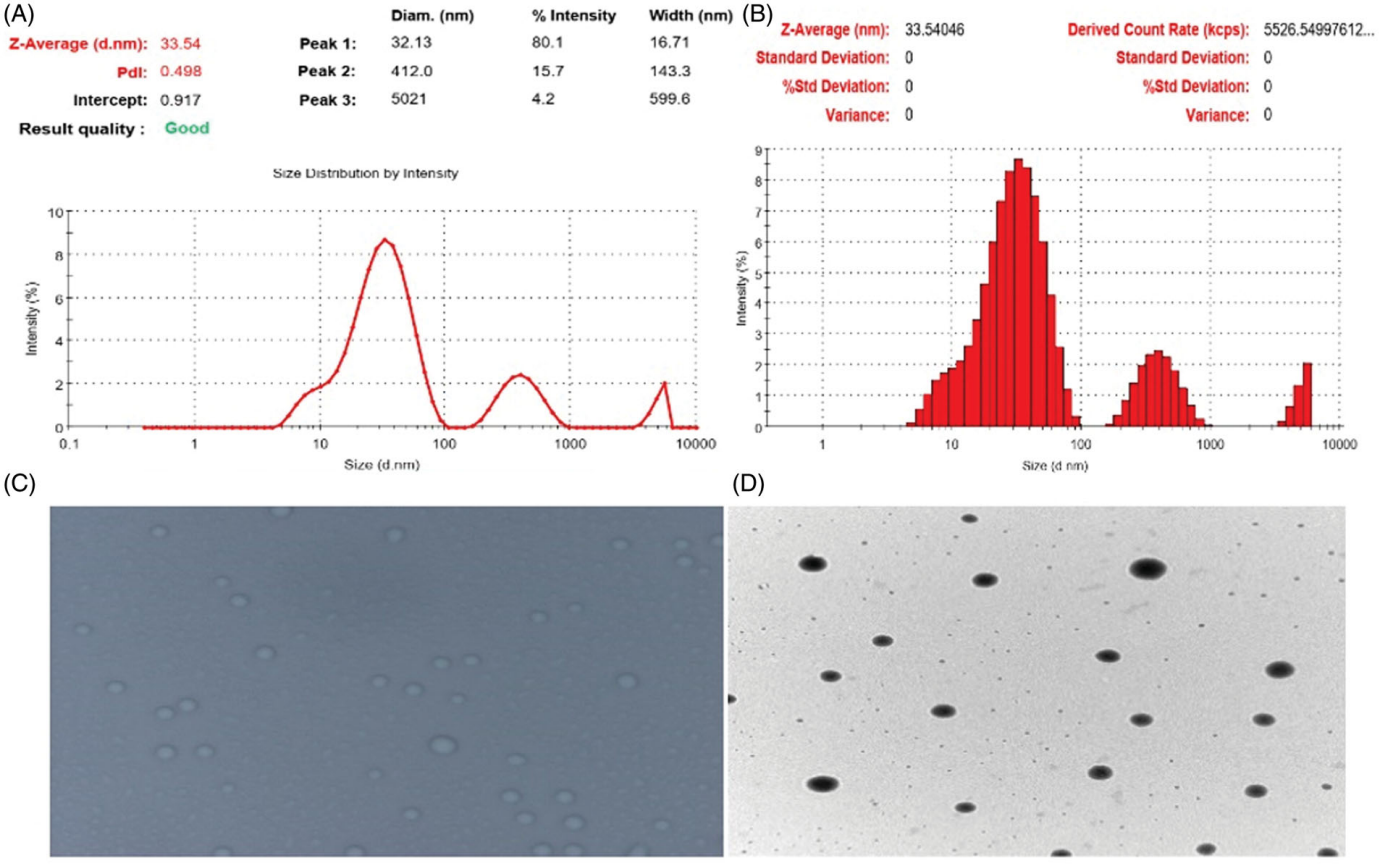
Characterization of optimized microemulsion (K7): (A, B) particle size distribution curve, (C) photo microscopy, and (D) transmission electron microscopy (TEM) analysis.

Microemulsion formulations of CRT loaded TTO ME based hydrogel, as well as their properties, were shown effective by the characterization study findings. When ME formulations were disseminated and studied macroscopic, they were found to be homogeneous, transparent with no precipitates, isotropic, and yellow colored. Whereas the physiochemical characteristics of various mentioned ME are depicted in Table 3. The drug content of the six specified formulations was in the range of 99.42–99.95% with a mean value of 99.63%. Furthermore, the developed ME's average size ranged from 33.54 to 80.12 nm, with PDI values ranging from 0.498 to 0.612. Furthermore, the enhanced drug content ensured that the drug was successfully loaded in various formulations. Although the zeta potential value was reported negative, which is nearly zero, this may be attributed to the presence of nonionic character of surfactant and a cosurfactant used (i.e. Cremophor^®^EL and Transcutol^®^P and phospholipid (PL90G)) whereas the presence of such elements provides better stability to the formulations. The mean diameter of optimized ME (K7), which was 33.54 nm, was found to be the smallest of all formulations (shown in Figure 2(A,B)). The zeta potential for the K7 was found to be 0.312 which could be due to the nonionic nature of the surfactant and co surfactant utilized. The RI value of the developed formulation was in the range of 1.318–1.412 against water. Therefore, this consistency in the RI of all formulations reflects the regular ME structure (Zhang et al., 2019).

**Table 3.** Various characteristics of selected microemulsion.

Microemulsion	Drug content (%)	Particle size (nm)	Zeta potential (mV)	RI
K7	99.42	33.54	0.312	1.321
K10	99.61	39.23	0.341	1.318
K12 (unstable)	99.53	–	–	1.51
K15	99.75	45.34	0.361	1.319
K13 (unstable)	99.95	–	0.412	1.412
K5	99.54	80.12	–	1.325

RI: refractive index.

### 3.4. Morphology and structure

Photomicrographs and TEMs were used to examine the appearance and structure of the optimized formulation (K7). Further, both the images depict the spherical nature of the globules (as shown in Figure 2(C,D)).

### 3.5. *In vitro* skin permeation studies

**Figure 3.**
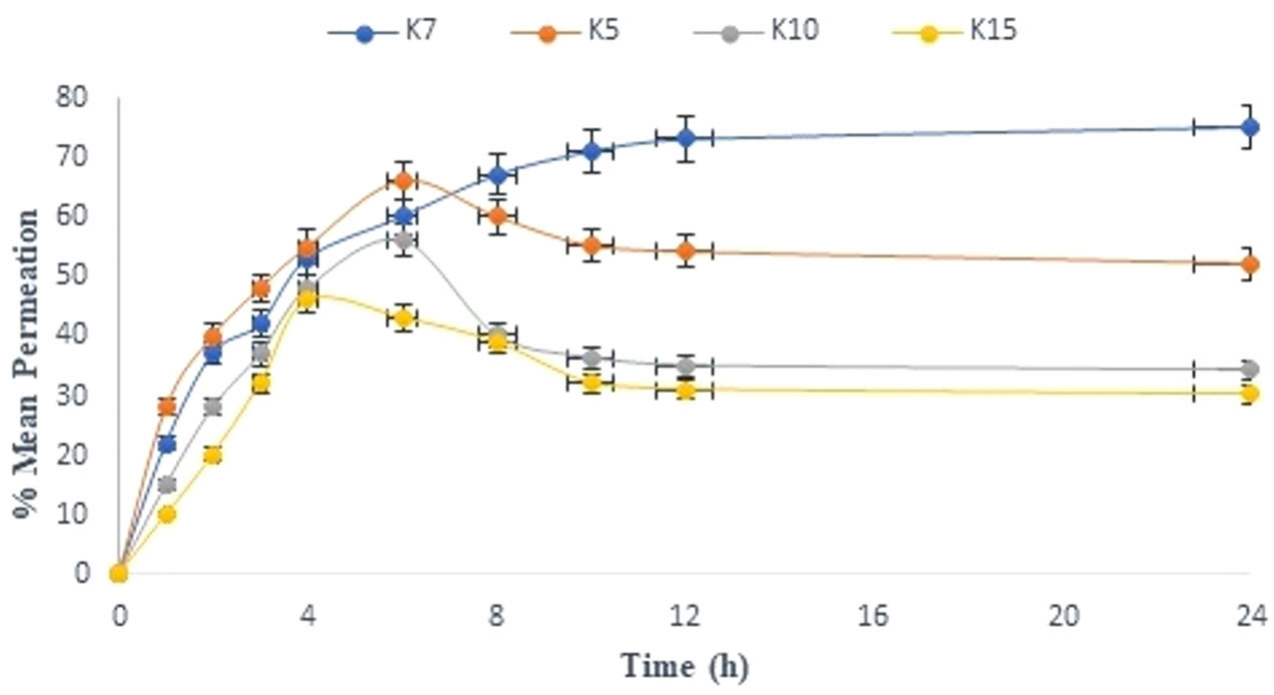
The percentage mean permeation vs. time for the various selected microemulsion. The average value ± SD (*n* = 3) is represented by each cross bar.

The permeation parameters of the given formulations are shown in Table 4. The permeation behavior of the mentioned formulations was analyzed by assessing the permeation flux as well as the graphic interpretation of the amount released with respect to time in Figure 3.

**Table 4.** Permeation profile and drug retention of CRT in epidermis from various formulations (*n* = 3).

Microemulsion code	Percentage of CRT permeated (%±SD)	Permeation flux (mg/cm^2^/h ± SD)	Drug retained (mg ± SD)
K7	75.21 ± 0.12	1.82 ± 0.012	0.861 ± 0.052
K10	56.32 ± 0.13	0.62 ± 0.01	0.512 ± 0.020
K15	46.32 ± 0.16	0.72 ± 0.02	0.491 ± 0.024
K5	66.21 ± 0.21	1.21 ± 0.011	0.664 ± 0.047

CRT: crotamiton.

The following sequence was observed for the amount of drug permeated from various treatment formulations:

K7 (75.21 ± 0.12%)>K5 (66.21 ± 0.21%)>K10 (56.32 ± 0.13%)>K15 (46.32 ± 0.16%)

The following was found to be the order of drug permeation flux:

$$K7\;\left(1.82\;\pm \;0.012\;{\text{μg }\text{cm}}^{-2}\;h^{-\;1}\right)>K5\;\left(1.21\;\pm \;0.011\;{\text{μg }\text{cm}}^{-2}\;h^{-\;1}\right)>K15\;\left(0.72\;\pm \;0.02\;{\text{μg }\text{cm}}^{-2}\;h^{-\;1}\right)>K10\;\left(0.62\;\pm \;0.01\;{\text{μg }\text{cm}}^{-2}\;h^{-\;1}\right)$$

It was observed that the rationale behind the amount of drug permeation with the K7 possesses higher amount of oil, irrespective of surfactant concentration, while the similar formulation K15 offered lowest permeation and relatively less permeation flux value. K5 has the next highest permeation flow after K7. Despite having the same phase diagram 1B, changes in composition and the amount of oil (16% in K7 vs. 14% in K5) are the causes of permeation profile variances. Surprisingly, both opposing observations were found to be common in this mixture. First, K5 have a maximum amount of *S*_mix_ (58%) over the K7 (*S*_mix_ 54%). However, the amount of phospholipid (PL) with K7 is (20.21%) and K15 (10.11%) observed highest and least in quantity, respectively, whereas in the case of K5, it was as found 14.32%; this came just after K7. As a result, it was deduced that the influence of PL on penetration was significantly greater than that of surfactant (Brand et al., 2002; Tabosa et al., 2018; Golwala et al., 2020). The reduced permeation flux in the K10 and K5 can be attributed to the lower concentration of phospholipid present. As a result, the presence of a lesser amount of phospholipid appears more important and deciding variable in determining the drug permeation profile than the growing amount of surfactant.

The cumulative effect of surfactant, PL, and a cosurfactant was found to have the greatest impact on drug permeation enhancement, followed by the amount of TTO and CRT. Previous studies reveal that surfactant quantity and type have a significant impact on the skin penetration of drug (Brand et al., 2002; Amiri-Rigi & Abbasi, 2019; Golwala et al., 2020).

### 3.6. Drug retention study

Table 4 represents the results of drug retention. This study was conducted after 24 h to determine the drug retention of four mentioned formulations. K7 has the greatest drug retention, after that K5 comes. As a result, the formulation K7 was chosen as the formulation for further study, due to the possession of the greatest permeation flux, the highest percentage permeation, and desirable drug retention (Radwan et al., 2017; Tabosa et al., 2018; Amiri-Rigi & Abbasi, 2019).

### 3.7. Comparative evaluation of the optimized ME formulation (K7)

#### 3.7.1. In vitro skin permeation study

**Figure 4.**
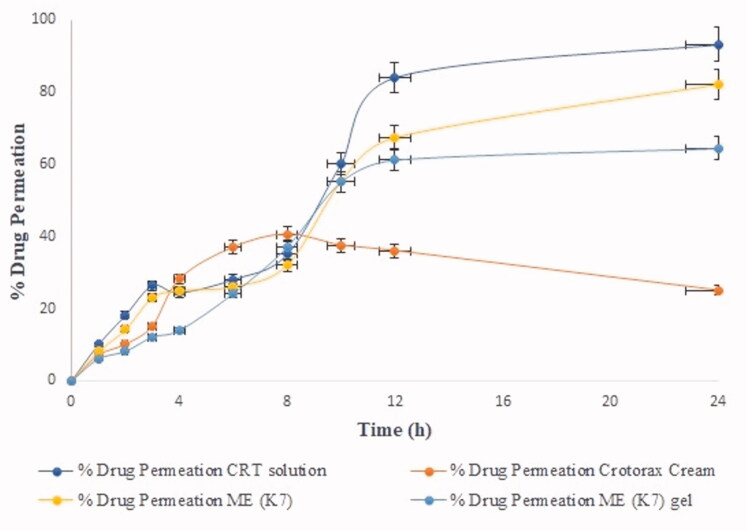
The percentage drug permeation of various mentioned formulation. The average value ± SD (*n* = 3) is represented by each cross bar.

*In vitro* skin permeation of ME (K7), ME (K7) hydrogel, commercial cream, and CRT solution through excised mice abdomen skin is illustrated in Figure 4. The release behavior of the aforementioned formulation was also evaluated by examining the flux value using a graphic representation.

The following is the order in which the release flux was found:

$$\text{Drug}\;\text{solution}\;\left(3.052\;{\text{mg}/\text{cm}}^{2}/h\;\pm \;0.02\right)>\text{ME}\;\left(2.34\;{\text{mg}/\text{cm}}^{2}/h\;\pm \;0.07\right)>\text{ME}\;\text{gel}\;\left(1.74\;{\text{mg}/\text{cm}}^{2}/h\;\pm \;0.002\right)>\text{conventional}\;\text{cream}\;\left(0.85\;{\text{mg}/\text{cm}}^{2}/h\;\pm \;0.011\right)$$

In the above order, the highest permeation flux was observed, followed by ME (K7) and ME (K7) hydrogel was observed higher over conventional cream (*p*<.05). The presence of higher concentration of surfactant and PL in ME revealed permeation enhancer and lowered the stratum corneum barrier. Apart from this, ME provides the larger surface area to contact with the upper layer of the skin and provides higher drug transport gradients to deeper epidermis, which is the driving force in topical drug delivery (Tabosa et al., 2018; Amiri-Rigi & Abbasi, 2019).

#### 3.7.2. Skin drug retention studies

**Figure 5.**
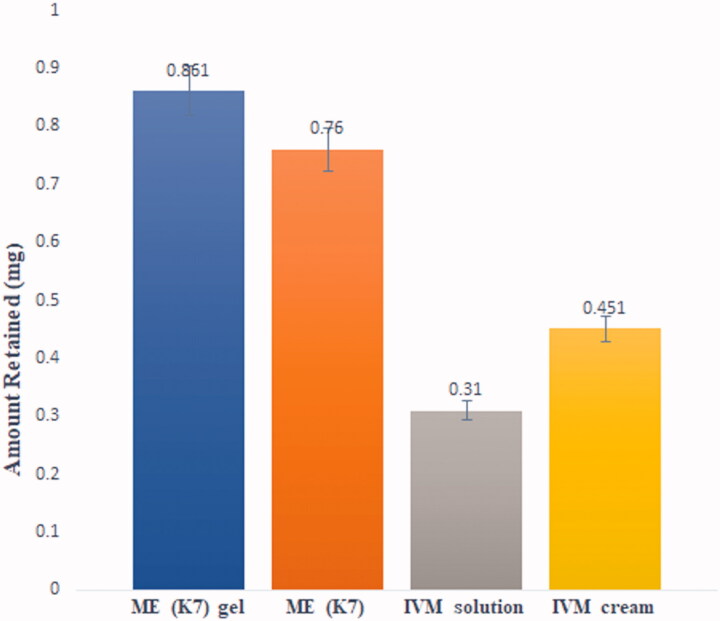
The drug retention in epidermis by various mentioned formulation. The average value ± SD (*n* = 3) is indicated by each cross bar.

The drug-retention experimental study compared the above-mentioned formulation as described above. The drug-retention profile of several CRT formulations is depicted in Figure 5. The optimized ME (K7) formulation found almost 2 and 2.5 times higher drug retention over the commercial CRT cream and CRT solution, respectively. Drug delivery may be enhanced owing to the greater drug penetration through the skin for the K7 formulation as opposed to the commercial cream and drug solution, resulting in more drug reaching it into the skin (Tabosa et al., 2018; Zhang et al., 2019). As concluded from the drug retention study, ME found the efficient delivery carrier and retained higher drug in epidermis, and provided effective therapy for treating scabies.

### 3.8. Dermatokinetic modeling

**Figure 6.**
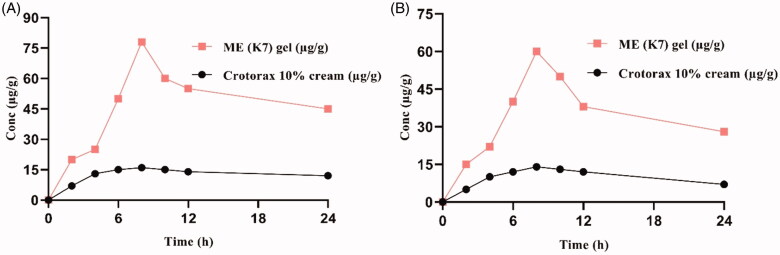
The drug concentration vs. time profile in (A) epidermis and (B) dermis at the different time points. Whereas each cross bar reflects the average value ± SD (*n* = 3).

The distribution of CRT in the epidermis and dermis of the mice skin is depicted in Figure 6(A,B). A significantly larger amount (*p*<.05) of CRT-loaded TTO was delivered in the skin layers using the ME gel (K7) than with conventional cream. The results from Table 5 show the quantitative values of AUC_0–12 h_, skin elimination rate constant (Ke), $T_{\text{max}}^{\text{Skin}},$ $C_{\text{max}}^{\text{Skin}},$ and skin penetration rate constant (*K*_p_). ME dramatically increased the biological half-life of the drug in both the epidermal and dermal layers, whereas *T*_max_ was greatly decreased, according to the findings of the investigations. However, *C*_max_ was enhanced in both the layers, and AUC was significantly enhanced in the dermis. The study findings revealed that ME (K7) gel may improve the delivery and topical bioavailability of CRT is higher in epidermis over the conventional product.

**Table 5.** Dermatokinetic parameters (mean ± SD) of ME (K7) gel in epidermis and dermis of mice (*n* = 6).

Dermatokinetic parameters	ME (K7) gel		Crotorax cream (10% w/w)	
	Epidermis	Dermis	Epidermis	Dermis
AUC_0–12 h_ (µg cm^–2^ h)	920 ± 340.12	718.12 ± 115.11	625.01 ± 130.21	505.11 ± 170
*C*_max_ (µg cm^–2^)	78.21 ± 2.8	58.32 ± 2.12	38.76 ± 5.1	40.01 ± 6.24
*T*_max_ (h)	3.12 ± 0.45	3.71 ± 0.34	6.81 ± 1.19	6.92 ± 0.68
*K*_a_ (h^–1^)	4.2 ± 230	4.3 ± 30.12	5.21 ± 24.02	4.9 ± 135.21
*K*_e_ (h^–1^)	4.7 ± 421.01	4.51 ± 32.03	6.93 ± 55.12	4.910 ± 135.6

### 3.9. Skin compatibility studies

**Figure 7.**
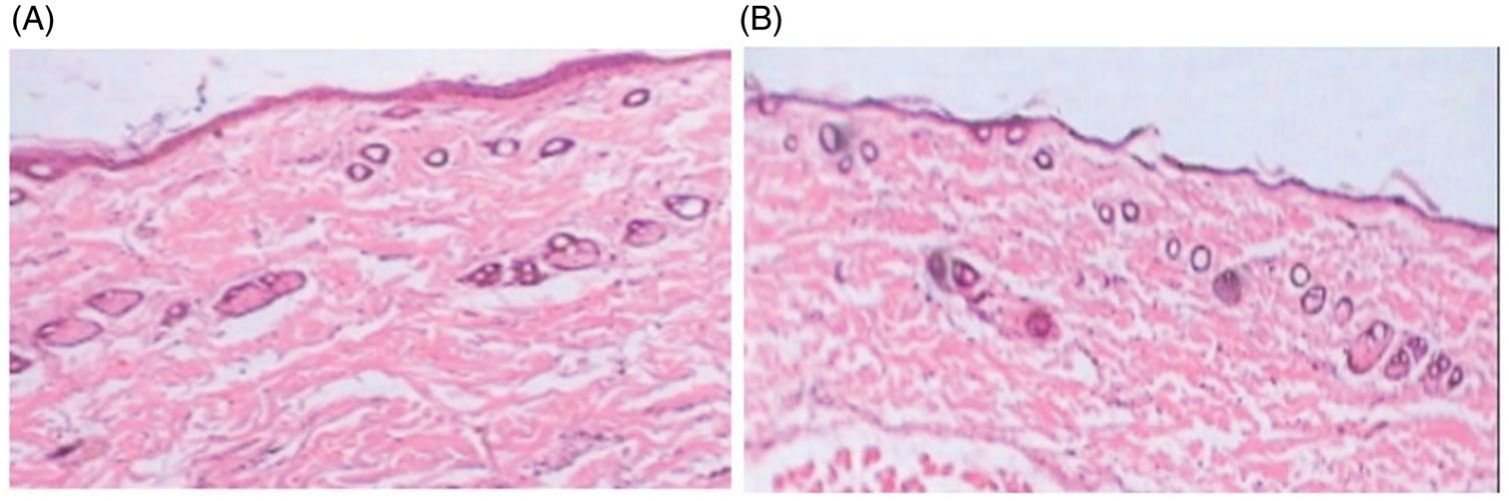
Histopathological microphotograph of skin treated with: (A) control (saline solution); (B) CRT loaded TTO ME (K7) hydrogel.

The histo-photomicrograph of the samples treated with ME (K7) hydrogel did not exhibit any drastic alterations, which demonstrate improved tolerability with the ME (K7) hydrogel, as shown in Figure 7(A,B). This may occur as a result of drug encapsulation inside biocompatible components such as phospholipids and surfactant. However, while Crotorax cream (10% w/w) (marketed by Abbott Healthcare Pvt. Ltd., Mumbai, India) caused inflammation in the mice skin, made the skin itchier and led to inflammatory cell infiltration, epidermis thickening, and epidermis peeling from the dermis are all indications of noncompliance (Brand et al., 2002; Tabosa et al., 2018).

## 4. Stability studies

The modified formulation's stability trials in terms of drug content **(**Table 6) and physical attributes (Table 7) were carried out at 4 ± 2 °C, 30 ± 2 °C, and 40 ± 2 °C for at least 6 weeks. After all, the specified week in Tables 6 and 7, the percentage drug content was calculated as per the method given in drug content tests of preliminary ME formulations. The optimized formulation was shown to be stable for six weeks at all of the tested temperatures, but the ME remained stable at 30 °C (for six weeks) and 40 °C (for almost 42 days). In these findings, it is shown that the improved ME may be maintained at room temperature, without the need to be refrigerated.

**Table 6.** Measurement of drug content of optimized formulation at different temperatures (*n* = 3).

S. no.	Days/ weeks	% drug content remaining		
		4 ± 2 °C	30 ± 2 °C	40 °C ± 2 °C
1.	1 day	99.61 ± 0.007	99.61 ± 0.007	99.61 ± 0.007
2.	1 week	99.53 ± 0.006	99.4899.61 ± 0.004	99.4199.61 ± 0.009
3.	2 weeks	99.4 ± 0.005	99.2199.61 ± 0.003	99.1099.61 ± 0.008
4.	4 weeks	99.21 ± 0.004	99.0199.61 ± 0.002	98.7899.61 ± 0.001
5.	5 weeks	99.13 ± 0.010	98.699.61 ± 0.001	98.5199.61 ± 0.003
6.	6 weeks	99.02 ± 0.007	98.51 ± 0.002	98.4199.61 ± 0.005
Net loss		0.59	1.1 ± 0.005	1.2 ± 0.002

**Table 7.** Physical characteristics of the optimized formulation.

S. no.	Days, weeks	Physical stability		
		4 ± 2 °C	30 ± 2 °C	40 °C ± 2 °C
1.	1 day	Pass	Pass	Pass
2.	1 week	Pass	Pass	Pass
3.	2 weeks	Pass	Pass	Pass
4.	4 weeks	Pass	Pass	Pass
5.	5 weeks	Pass	Pass	Pass
6.	6 weeks	Pass	Pass	Pass
Net result		>42 days (6 weeks)	>42 days (6 weeks)	>42 days (6 weeks)

## 5. Conclusions

It may claim that CRT loaded TTO ME-based hydrogel with huge potential exists for fighting the issues that occur with the current drug. In this research, the ME-based hydrogel showed significantly increased epidermal deposition for CRT, and improved skin targeting properties compared to the commercial formulation. The ME converted hydrogel demonstrated the capacity to release the drug slowly with minimal discomfort, making it possible for clinical use. Dermatokinetic modeling revealed that the epidermal layer of the skin has an appropriate concentration of drugs. Overall, the results show that a nanosized TTO ME-based hydrogel system may enhance CRT in a site-specific manner.
